# Epidemiologic Trends of and Factors Associated With Overall Survival for Patients With Gastroenteropancreatic Neuroendocrine Tumors in the United States

**DOI:** 10.1001/jamanetworkopen.2021.24750

**Published:** 2021-09-23

**Authors:** Zihan Xu, Li Wang, Shuang Dai, Mingjing Chen, Feng Li, Jianguo Sun, Feng Luo

**Affiliations:** 1 Lung Cancer Center, Cancer Center and State Key Laboratory of Biotherapy, West China Hospital of Sichuan University, Chengdu, Sichuan, China; 2 Cancer Institute of People’s Liberation Army, Xinqiao Hospital, Army Medical University, Chongqing, People’s Republic of China

## Abstract

**Importance:**

Although the incidence and prevalence of gastroenteropancreatic neuroendocrine tumors (GEP-NETs) have been thought to have increased during the past decades, updated epidemiologic and survival data are lacking.

**Objectives:**

To conduct an epidemiologic and survival analysis of the largest cohort of patients with GEP-NETs using the latest data and to establish a novel nomogram to predict the survival probability of individual patients with GEP-NETs.

**Design, Setting, and Participants:**

In this cohort study, 43 751 patients with GEP-NETs diagnosed from January 1, 1975, to December 31, 2015, were identified from the Surveillance, Epidemiology, and End Results Program. Associated data were used for epidemiologic and survival analysis, as well as the establishment and validation of a nomogram to predict the survival probability of individual patients with GEP-NETs. The study cutoff date was December 31, 2018. Statistical analysis was performed from February 1 to April 30, 2020.

**Main Outcomes and Measures:**

Incidence, factors associated with overall survival, and a nomogram model for patients with GEP-NETs.

**Results:**

A total of 43 751 patients received a diagnosis of GEP-NETs from 1975 to 2015 (22 398 women [51.2%], 31 976 White patients [73.1%], 7097 Black patients [16.2%], 3207 Asian and Pacific Islander patients [7.3%], 270 American Indian and Alaska Native patients [0.6%], and 4546 patients of unknown race [10.4%]; mean [SD] age at diagnosis, 58 [15] years). The age-adjusted incidence rate of GEP-NETs increased 6.4-fold from 1975 to 2015 (annual percentage change [APC], 4.98; 95% CI, 4.75-5.20; *P* < .001). Furthermore, among site groups, the incidence of GEP-NETs in the rectum increased most significantly (APC, 6.43; 95% CI, 5.65-7.23; *P* < .001). As for stage and grade, the incidence increased the most in localized GEP-NETs (APC, 6.53; 95% CI, 6.08-6.97; *P* < .001) and G1 GEP-NETs (APC, 18.93; 95% CI, 17.44-20.43; *P* < .001). During the study period, the mean age at diagnosis for localized disease increased by 9.0 years (95% CI, 3.3-14.7 years; *P* = .002), which remained unchanged for regional and distant cases. On multivariable analyses, age, sex, marital status, and tumor size, grade, stage, and site were significantly associated with overall survival for patients with GEP-NETs (eg, patients with distant vs localized disease: hazard ratio, 10.32; 95% CI, 8.56-12.43; G4 vs G1 GEP-NET: hazard ratio, 6.37; 95% CI, 5.39-7.53). Furthermore, a nomogram comprising age, size, grade, stage, and site was established to predict the 3-year and 5-year survival probability, with the concordance indexes of 0.893 (95% CI, 0.883-0.903) for the internal validations and 0.880 (95% CI, 0.866-0.894) for the external validations. The receiver operating characteristic curve demonstrated that the nomogram exhibited better discrimination power than TNM classification (area under the curve for 3-year overall survival, 0.908 vs 0.795; for 5-year overall survival, 0.893 vs 0.791).

**Conclusions and Relevance:**

In this study, the incidence and prevalence of GEP-NETs have continued to increase over 40 years, especially among patients with rectal GEP-NETs. In addition, this study suggests that a nomogram with 5 prognostic parameters may accurately quantify the risk of death among patients with GEP-NETs, indicating that it has satisfactory clinical practicality.

## Key Points

**Question:**

What are the epidemiologic trends of and prognostic factors for gastroenteropancreatic neuroendocrine tumors (GEP-NETs) in patients in the US?

**Findings:**

In this cohort study of 43 751 patients with GEP-NETs, most cases occurred in the rectum and small intestine. The age-adjusted incidence rate of GEP-NETs increased significantly from 1975 to 2015, especially for rectal, localized, and grade 1 GEP-NETs; age, sex, marital status and tumor size, grade, stage, and site were significantly associated with overall survival for patients with GEP-NETs.

**Meaning:**

The incidence and prevalence of GEP-NETs have continued to increase over 40 years; this study suggests that a predictive model based on prognostic factors may accurately quantify the risk of death among patients with GEP-NETs, indicating that the model has satisfactory clinical practicality.

## Teaser

This cohort study uses data from the Surveillance, Epidemiology, and End Results Program to conduct an epidemiologic and survival analysis of patients with gastroenteropancreatic neuroendocrine tumors and to establish a nomogram to predict the survival probability of these patients.

## Introduction

Neuroendocrine tumors (NETs) are a heterogeneous class of rare cancers with different biological behaviors that arise from cells throughout the diffuse endocrine system. Because approximately two-thirds of NETs occur in the gastroenteropancreatic system, which mainly includes the stomach, small intestine, colon, appendix, rectum, and pancreas, gastroenteropancreatic NETs (GEP-NETs) are the main subtype of NETs.^1,2,3^ Although the biological behavior of some tumors is relatively indolent, other GEP-NETs may be more aggressive and associated with poor prognosis.^4^

The increasing number of articles published each year on NETs proves that global attention to NETs has been increasing, which might be due to the reported increasing incidence.^5,6,7^ However, to our knowledge, there is a lack of updated data on the epidemiologic characteristics and on the survival analysis of patients with GEP-NETs. On the other hand, given the rarity and indolent biological behavior of GEP-NETs, most studies on GEP-NETs are based on a small (or very small) number of cases in single institutions.^8,9,10,11^ In addition, population-based studies have never specifically targeted GEP-NETs but rather only addressed the issue of NETs in a single gastrointestinal tract site (eg, stomach,^12^ colon,^13^ small intestine,^14^ appendix,^15^ or pancreas^16^). Therefore, in this study, we performed a population-based study using the information in the National Cancer Institute’s Surveillance, Epidemiology, and End Results (SEER) Program to systematically analyze the epidemiologic, clinical, and prognostic characteristics of GEP-NETs.

Because of the complex and inconsistent treatment of GEP-NETs, the prognosis of patients with GEP-NETs is still difficult to assess. To date, the prognostic analysis of NETs is still based mainly on the American Joint Committee on Cancer and the European Neuroendocrine Oncology TNM staging system. This system evaluates patients’ prognosis based on tumor volume (T), regional lymph node tumor involvement (N), and distant metastases (M). However, other basic clinicopathologic characteristics, such as age, sex, race and ethncity, and tumor grade and location can also be associated with patients’ prognosis.^17^ Among the currently available prediction tools, nomograms are considered to be the most accurate and characteristic method for predicting the prognosis of patients with cancer.^18^ For certain malignant neoplasms, patients’ mortality risk has been successfully quantified by combining relevant prognostic factors.^19,20,21^ However, to our knowledge, few studies have used nomograms to predict the prognosis of patients with GEP-NETs. This study sought to develop a more detailed nomogram based on the relatively large cohort of patients with GEP-NETs in the SEER database to predict 3-year and 5-year overall survival (OS).

## Methods

### Data Source

The SEER database, which is an authoritative source of information on cancer epidemiology (incidence and prevalence) and clinical characteristics (primary tumor site, tumor morphologic characteristics and stage at diagnosis, first course of treatment, and follow-up for vital status) in the United States, was used for this study. Race and ethnicity were self-reported. Patients were divided into the 4 following categories: White, Black, Asian and Pacific Islander, and American Indian and Alaska Native. The study design is presented in eFigure 1 in the Supplement. We used histologic codes from the *International Classification of Diseases for Oncology, Third Edition*, and site codes to identify patients who received a diagnosis of GEP-NETs from January 1, 1975, to December 31, 2015 (eTable 1 in the Supplement). The West China Hospital of Sichuan University institutional review board deemed this study exempt from review and informed consent because the data used in the study are freely available. This study followed the Strengthening the Reporting of Observational Studies in Epidemiology (STROBE) reporting guideline.

### Stage and Classification of GEP-NETs

Because of the lack of a unified and reasonable staging system for GEP-NETs, the SEER staging system was used in our research. Overall, tumors were staged as localized, regional, or distant diseases. Localized GEP-NETs were defined as aggressive tumors that were completely confined to the organ of origin. Regional GEP-NETs were defined as neoplasms that extended (1) beyond the limits of the organ of origin directly into surrounding organs or tissues, (2) into regional lymph nodes by way of the lymphatic system, or (3) by a combination of extension and regional lymph nodes. Finally, distant GEP-NETs were defined as tumors that spread to areas of the body that are far from the primary tumor or away from the primary tumor. For tumor classification, the SEER classification scheme systematically classifies the cases into 4 grades: grade 1 (G1), with high discrimination; G2, moderately differentiated; G3, poorly differentiated; and G4, undifferentiated or anaplastic.

### Nomogram Construction and Validation

All eligible patients (n = 13 515) were randomly divided into 2:1 training (n = 9010) and validating (n = 4505) groups. Multivariable Cox proportional hazards regression models were used for evaluating factors associated with OS. The verification of the nomogram is based mainly on the internal (training cohort) and external (validation cohort) discrimination and calibration measurements. The consistency index (C index), which mainly measures the differences between the predicted and actual outcomes, is the main index used to evaluate the discriminative ability of the nomogram. The calibration curve was used to compare predicted survival according to the nomogram and actual survival. Furthermore, the accuracy of the nomogram and TNM staging system for 3-year and 5-year survival prediction was compared by using the area under the receiver operating characteristic curve (AUC).

### Statistical Analysis

Statistical analysis was performed from February 1 to April 30, 2020. Descriptive statistics *t* tests or χ^2^ tests were used to compare patients’ basic clinical characteristics as follows: year of diagnosis, age, sex, race and ethnicity, marital status, tumor size, tumor grade, tumor stage, treatment, and primary tumor sites.

The age-adjusted incidence and limited-duration prevalence rates (10-year and 20-year rates) were calculated by the SEER*Stat software, version 8.3.8 (Surveillance Research Program, National Cancer Institute). The annual percentage change (APC) was calculated by fitting a simple linear model; the logarithm of the yearly age-adjusted rates first is regressed on time, then a transformation of the slope is used to calculate the percentage change per year.^22^ The APCs were comparable across scales, allowing for comparisons of incidences between cohorts with rare and common malignant neoplasms.^23^

The outcome of interest was cancer-specific mortality, extracted from the variable cause-specific death classification. The SEER*Stat program estimates survival time by subtracting the date of diagnosis from the date of last follow-up (the study cutoff). The study cutoff date was December 31, 2018. We included patients diagnosed only from 1975 to 2015 to guarantee that all included patients could be observed for more than 3 years. Cancer-specific mortality is represented by Kaplan-Meier survival curves and compared using the log-rank test.

Cox proportional hazards multivariable regression was used to evaluate the association of age, sex, race and ethnicity, marital status, tumor size, disease stage, and tumor site with OS by calculating hazard ratios (HRs) and 95% CIs with other factors adjusted. Statistical analyses were conducted with SPSS, version 23 (IBM Corp). All *P* values were from 2-sided tests, and results were deemed statistically significant at *P* < .05.

## Results

### Patient Characteristics

From 1975 to 2015, a total of 43 751 patients (mean [SD] age at diagnosis, 58 [15] years; median age, 58 years [range, 2-100 years]) with GEP-NETs were identified from the SEER database. Among them, the ratio of men (21 353 [48.8%]) to women (22 398 [51.2%]) was close to 1 (eTable 2 in the Supplement). Further, 73.1% (31 976) were White patients, 16.2% (7097) were Black patients, 7.3% (3207) were Asian and Pacific Islander patients, 0.6% (270) were American Indian and Alaska Native patients, and 10.4% (4546) were patients with unknown race. Of 18 733 GEP-NETs (42.8%) with a known grade, 12 740 (29.1%) were G1, 3255 (7.4%) were G2, 2091 (4.8%) were G3, and 647 (1.5%) were G4. Among 40 280 GEP-NETs (92.1%) with a known stage, 22 502 (51.4%) were localized, 8199 (18.7%) were regional, and 9579 (21.9%) were distant disease at the time of diagnosis. As for primary tumor sites of GEP-NETs, the rectum was most commonly involved (12 532 [28.6%]), followed by the small intestine (12 285 [28.1%]) and pancreas (7167 [16.4%]). Gastric sites accounted for 9.2% (4040), colonic sites for 9.2% (4010), and appendiceal sites for 8.5% (3717).

### Annual Incidence

**Figure 1.**
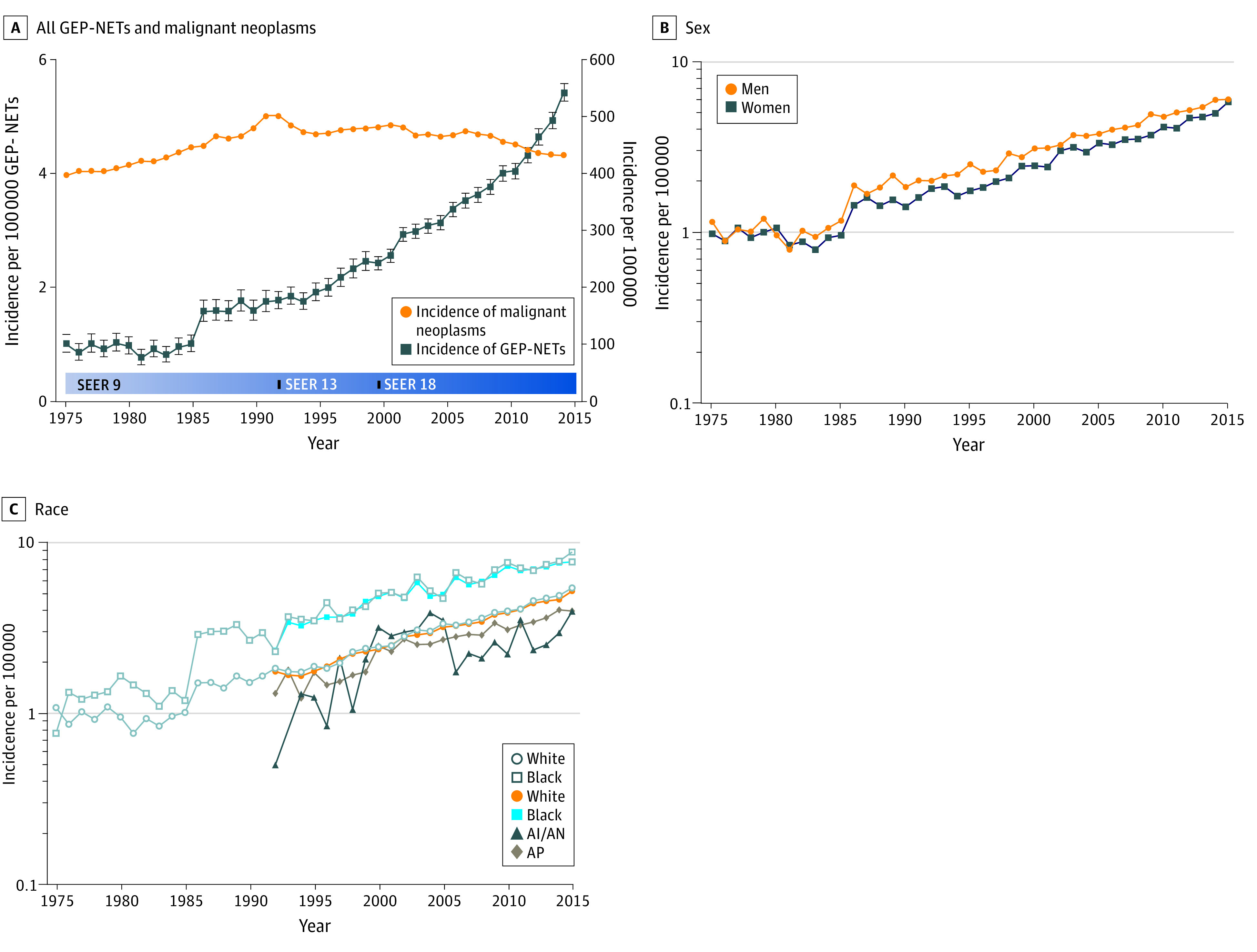
Incidence of Gastroenteropancreatic Neuroendocrine Tumors (GEP-NETs) Over Time by Sex and Race A, Annual age-adjusted incidence of all GEP-NETs by year (1975-2015). The incidence is presented as the number of tumors per 100 000 population (with 95% CIs presented as whiskers) age-adjusted for the 2000 US standard population. B, Time-trend analyses of the incidence of GEP-NETs by sex (1975-2015). C, The incidence of GEP-NETs by race (1975-2015), including expanded racial categories (1992-2015). AI/AN indicates American Indian and Alaska Native; AP, Asian and Pacific Islander; and SEER, Surveillance, Epidemiology, and End Results Program.

Using population data derived from the SEER program, we calculated the age-adjusted incidence of GEP-NETs per 100 000 individuals per year, referring to the 2000 US standard population. The overall age-adjusted incidence of GEP-NETs was 1.05 per 100 000 persons in 1975, which increased to 5.45 per 100 000 persons by 2015, with an APC of 4.98 (95% CI, 4.75-5.20), as shown in Figure 1A (contrasted with the annual age-adjusted incidence of all malignant neoplasms [APC, 0.19; 95% CI, 0.04-0.35]). Detailed incidence data are presented in eTable 3 in the Supplement. With regard to the sex of patients with GEP-NETs, the age-adjusted incidence rates of male and female patients significantly increased over time, with an APC of 4.90 (95% CI, 4.64-5.15) for male patients and 4.99 (95% CI, 4.71-5.27) for female patients (Figure 1B). The long-term trend of the incidence of GEP-NETs in different races and ethnicities can be explored using the data from SEER 9. Incidence rates among White and Black populations increased until 2015 (White populations: APC, 4.84 [95% CI, 4.60-5.08]; Black populations: APC, 4.68 [95% CI, 4.20-5.16]). Because SEER 13 (1992-2015) recorded more detailed racial and ethnic categories, we can further explore these trends (Figure 1C). Among White, Black, Asian and Pacific Islander populations, incidence rates of GEP-NETs increased during the period from 1992 to 2015 (White populations: APC, 5.02 [95% CI, 4.81-5.23]; Black populations: APC, 4.07 [95% CI, 3.55-4.59]; Asian and Pacific Islander populations: APC, 4.52 [95% CI, 3.91-5.13]), whereas rates for the American Indian and Alaska Native population (APC, 2.24 [95% CI, −0.10 to 4.64]) remained unchanged.

**Figure 2.**
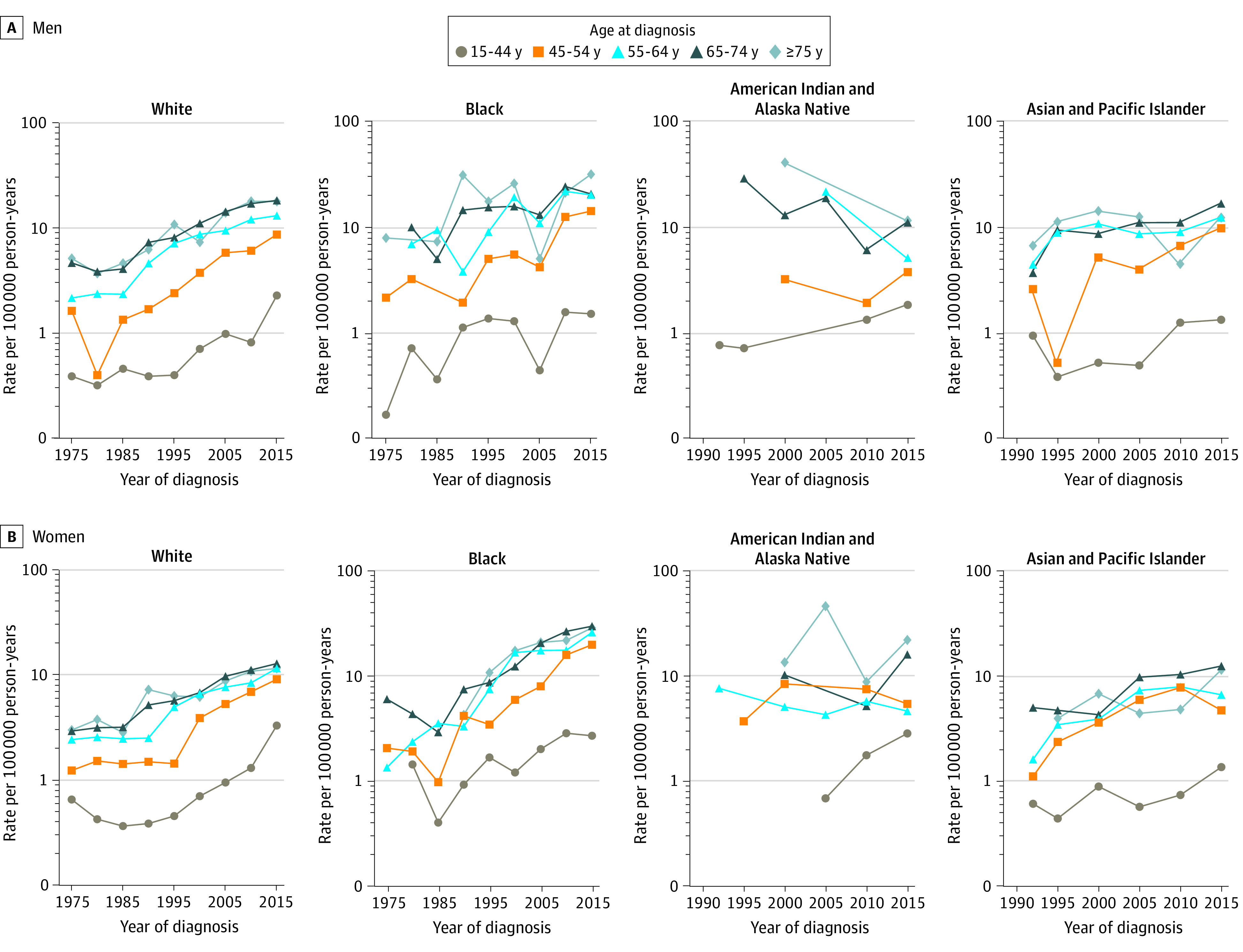
Gastroenteropancreatic Neuroendocrine Tumors (GEP-NETs) Incidence Trends by Sex, Race, and Age at Diagnosis, Surveillance, Epidemiology, and End Results Program 9 (1975-1991) and 13 (1992-2015)

The increasing incidence of GEP-NETs in the White population was associated with increases among all age groups (15-44, 45-54, 55-64, 65-74, and ≥75 years) for male patients (15-44 years: APC, 5.42 [95% CI, 4.74-6.11]; 45-54 years: APC, 6.01 [95% CI, 5.36-6.66]; 55-64 years: APC, 4.81 [95% CI, 4.39-5.23]; 65-74 years: APC, 4.34 [95% CI, 3.92-4.76]; ≥75 years: APC, 3.99 [95% CI, 3.37-4.61]) and female patients (15-44 years: APC, 5.15 [95% CI, 4.18-6.12]; 45-54 years: APC, 5.84 [95% CI, 5.22-6.46]; 55-64 years: APC, 4.88 [95% CI, 4.42-5.34]; 65-74 years: APC, 4.00 [95% CI, 3.66-4.33]; ≥75 years: APC, 3.41 [95% CI, 2.88-3.94]) (Figure 2). Among the Black population, rates increased for only 1 age group—women aged 45 to 54 years (APC, 5.59 [95% CI, 4.67-6.52]). The increasing rates among the Asian and Pacific Islander population were due to increases among all age groups except those aged ≥75 years (15-44, 45-54, 55-64 and 65-74 years) for male patients (15-44 years: APC, 4.00 [95% CI, 1.63-6.43]; 45-54 years: APC, 5.72 [95% CI, 3.93-7.54]; 55-64 years: APC, 2.25 [95% CI, 0.83-3.69]; 65-74 years: APC, 4.69 [95% CI, 2.81-6.60]) and female patients (15-44 years: APC, 4.42 [95% CI, 2.84-6.03]; 45-54 years: APC, 4.89 [95% CI, 3.16-6.65]; 55-64 years: APC, 4.76 [95% CI, 2.71-6.86]; 65-74 years: APC, 4.53 [95% CI, 2.12-7.00]) (Figure 2).

### Incidence and Prevalence According to Tumor Site, Stage, and Grade

**Figure 3.**
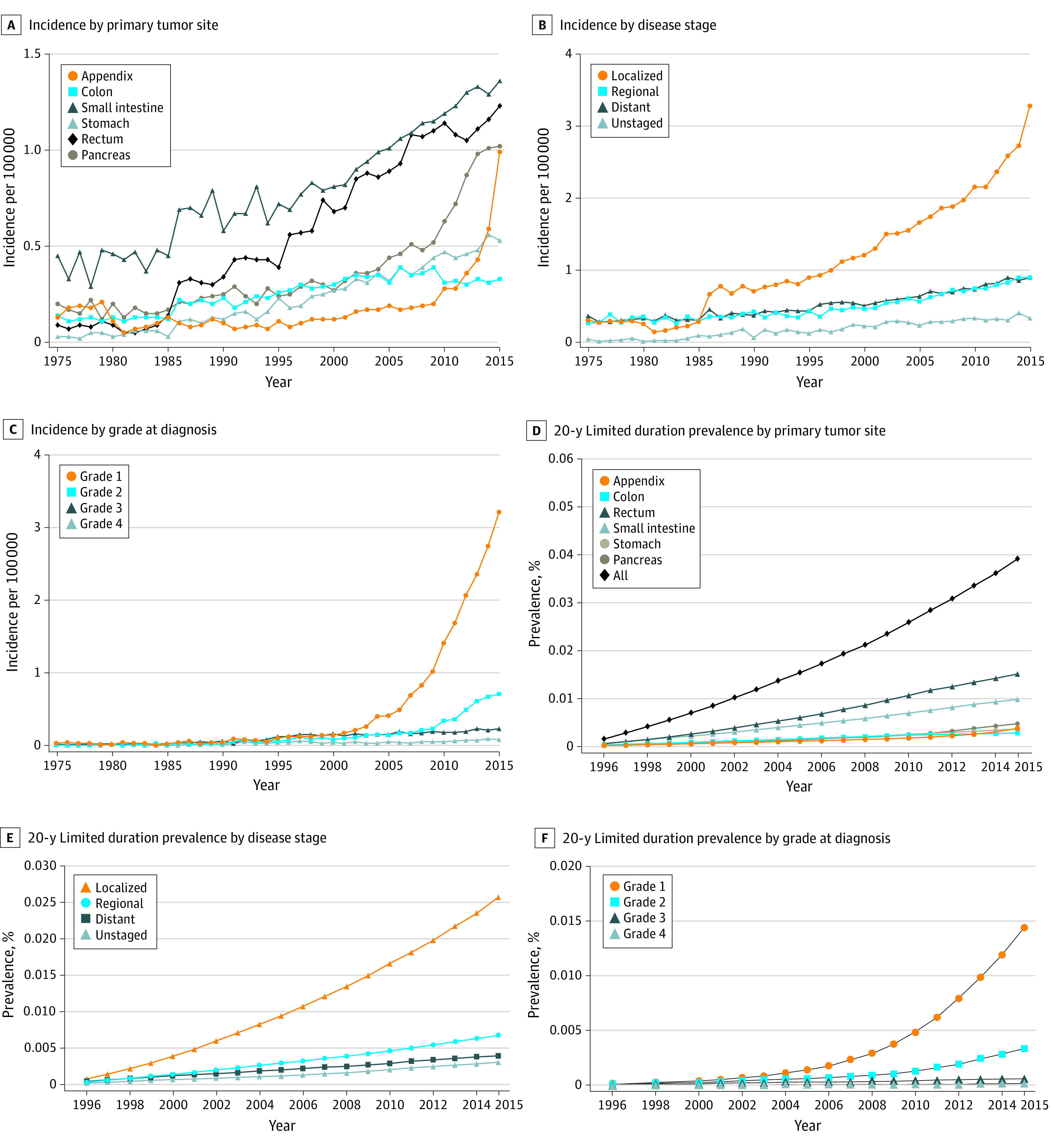
Incidence and Limited Duration Prevalence of Gastroenteropancreatic Neuroendocrine Tumors (GEP-NETs) Over Time by Site, Disease Stage, and Grade

The incidence of GEP-NETs continued to increase during the analyzed period; this change is widely reflected in different GEP-NET primary sites, stages, and grades. The APCs for various sites ranged from 2.87 (95% CI, 2.28-3.47) in the colon to 6.43 (95% CI, 5.65-7.23) in the rectum (Figure 3A). Among stage groups, the incidence of localized GEP-NETs increased the most, from 0.32 per 100 000 persons in 1975 to 3.28 per 100 000 persons in 2015 (APC, 6.53; 95% CI, 6.08-6.97; *P* < .001) (Figure 3B). As for different grades, the incidence increased the most for G1 GEP-NETs, from 0.03 per 100 000 persons in 1975 to 3.50 per 100 000 persons in 2015 (APC, 18.93; 95% CI, 17.44-20.43; *P* < .001) (Figure 3C).

In addition, the 20-year limited-duration prevalence of GEP-NETs increased significantly from 0.00138% in 1996 to 0.03917% in 2015, which also reflected the increasing incidence and indolence characteristics (*P* < .001) (Figure 3D). The detailed 20-year and 10-year limited-duration prevalence and absolute counts are listed in eTable 4 in the Supplement. As for different primary tumor sites, the prevalence of rectal GEP-NETs (0.01505% in 2015) was the highest, then followed by the small intestine (0.00974% in 2015) (Figure 3D). Among stage groups, prevalence increased the most in localized disease (from 0.00066% in 1996 to 0.02566% in 2015), followed by regional disease (from 0.00024% in 1996 to 0.00667% in 2015) (Figure 3E). As for different tumor grades, prevalence increased the most in G1 GEP-NETs (from 0.00009% in 1996 to 0.01435% in 2015) (Figure 3F).

### Age at Diagnosis Trends

We next calculated the mean age at diagnosis of patients with GEP-NETs yearly from 1975 to 2015 by stage (eFigure 2A in the Supplement). During the 41-year study period, the mean age at diagnosis for localized cases increased by 9.0 years (95% CI, 3.3-14.7 years; *P* = .002). In contrast, the mean age at diagnosis for regional and distant cases remained unchanged. The trajectory for the mean age during the period for localized stage was significantly different compared with the mean age for regional and distant cases (localized vs regional: mean difference, −5.914; 95% CI, −7.262 to −4.567; *P* < .001; localized vs distant: mean difference, −6.439; 95% CI, −7.962 to −4.915; *P* < .001).

### Survival

The median OS for all patients was 324 months (range, 320.7-330.6 months). Patients with rectal (median OS, not reached [NR]) or appendiceal (median OS, NR) GEP-NETs had the best median OS among site groups, whereas patients with GEP-NETs in the pancreas (67 months; range, 62.5-71.5 months) had the worst median OS (eFigure 3A in the Supplement). As for the different age groups, we found that patients aged 60 years or older had the worst median OS (262 months; range, 235.0-289.0 months) compared with patients younger than 60 years (median OS, NR) (eFigure 3B in the Supplement). Patients with localized GEP-NETs had better median OS (median OS, NR) compared with those with regional GEP-NETs (297 months; range, 242.4-351.6 months) or distant GEP-NETs (34 months; range, 32.2-35.8 months) (eFigure 3C in the Supplement). When we calculated survival by tumor grade, we found that patients with G3 GEP-NETs and patients with G4 GEP-NETs had nearly identical survival curves; the median OS of patients with G3 GEP-NETs was 11 months (range, 9.9-12.0 months), and the median OS of patients with G4 GEP-NETs was 9 months (range, 7.7-10.3 months). However, the median OS of patients with G1 or G2 GEP-NETs was not reached, which was significantly better than high-grade (G3 and G4) cases (eFigure 3D in the Supplement). All these survival analyses were statistically significant (*P* < .001).

We further evaluated the survival patterns according to site and stage (eTable 5 in the Supplement). For most sites, the median OS for patients with localized GEP-NET was not reached, except for those with GEP-NETs of the colon (402 months; range, 207.6-596.5 months). For patients with regional GEP-NETs, the median OS for those with GEP-NETs in the stomach was the worst (100 months; range, 30.9-169.1 months), whereas patients with GEP-NETs in the appendix had the best median OS (NR). For patients with distant GEP-NETs, the median OS for those with GEP-NETs in the small intestine was the best (96 months; range, 89.0-103.0 months); patients with GEP-NETs in the colon (8 months; range, 6.8-9.2 months), stomach (9 months; range, 7.3-10.7 months), or rectum (11 months; range, 8.8-13.2 months) had the worst median OS. We also evaluated the 3-year and 5-year survival rates according to site and stage. Both 3-year and 5-year survival rates for patients with local disease were more than 90%. Even for regional GEP-NETs in some sites, substantial rates of 3-year and 5-year survival were noted, ranging from 55.6% (SE: 3.3%) at 3 years and 52.8% (SE: 3.3%) at 5 years for GEP-NETs in the stomach to 93.3% (SE: 0.4%) at 3 years and 89.2% (SE: 0.5%) at 5 years for GEP-NETs in the small intestine. At all sites, survival for patients with distant stage GEP-NETs was poor, with the exception of GEP-NETs in the small intestine, for which the 3-year survival rate was 74.9% (SE: 0.8%) and the 5-year survival rate was 63.2% (SE: 0.9%).

Overall, 3-year and 5-year OS for regional stage cases improved from 75.8% (SE: 8.0%) at 3 years and 75.8% (SE: 8.0%) at 5 years in 1975 to 94.8% (SE: 0.9%) at 3 years and 94.8% (SE: 0.9%) at 5 years in 2015. Large improvements in 3-year and 5-year survival were also observed for distant cases (from 48.3% [SE: 8.3%] at 3 years and 39.5% [SE: 8.8%] at 5 years in 1975 to 60.9% [SE: 2.1%] at 3 years and 60.9% [SE: 2.1%] at 5 years in 2015), and more modest improvements were seen for localized stage cases (from 96.4% [SE: 3.5%] at 3 years and 92.7% [SE: 5.0%] at 5 years in 1975 to 98.4% [SE: 0.4%] at 3 years and 98.4% [SE: 0.4%] at 5 years in 2015) (eFigure 2B and C in the Supplement).

### Multivariable Analysis of OS

We next performed multivariable analysis to further explore the independent prognostic risk factors for patients with GEP-NETs according to the Cox proportional hazards regression model. We included potentially prognostic parameters, such as age, sex, race and ethnicity, marital status, tumor size, tumor grade, disease stage, and primary tumor site in this model.

**Figure 4.**
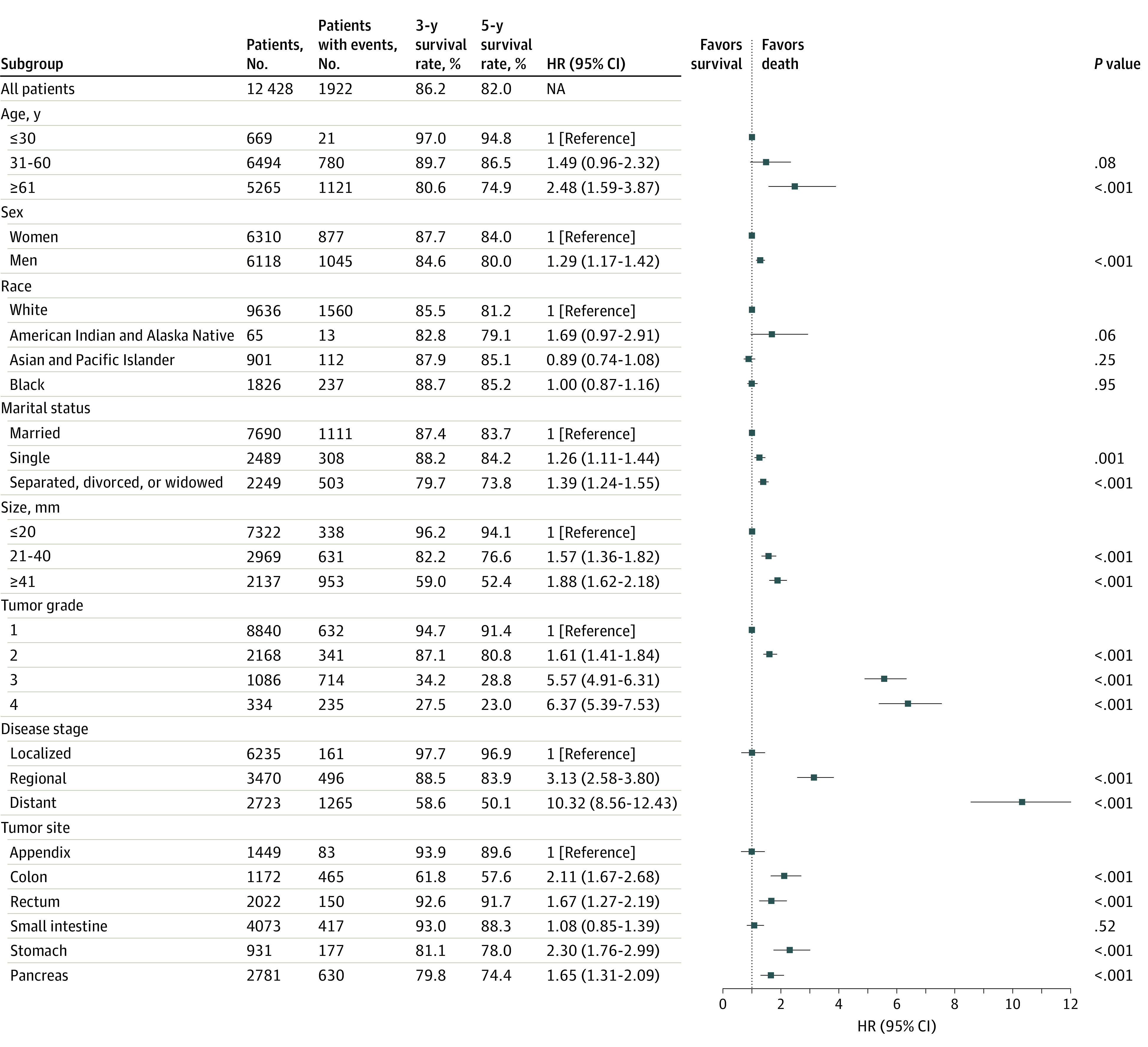
Multivariable Regression Analysis for Gastroenteropancreatic Neuroendocrine Tumors (GEP-NETs) HR indicates hazard ratio.

We found that disease stage was the most important risk factor for prognosis. Compared with localized disease, the risk of death for distant disease was greatly increased (HR, 10.32; 95% CI, 8.56-12.43). Except for race and ethnicity, the other parameters, including age, sex, marital status, tumor size, tumor grade, and tumor site, were all found to be significantly associated with survival. Overall survival was poorer for patients with G3 (HR, 5.57; 95% CI, 4.91-6.31) or G4 (HR, 6.37; 95% CI, 5.39-7.53) GEP-NETs than for patients with G1 GEP-NETs, after adjustment for other covariates. When we used appendiceal GEP-NETs as a reference, we observed that patients with primary GEP-NETs in the small intestine (HR, 1.08; 95% CI, 0.85-1.39; *P* = .52) showed no significant difference in survival. By contrast, the patients with GEP-NETs in other primary sites all showed poorer survival than patients with appendiceal GEP-NETs. Among them, patients with GEP-NETs in the stomach had the worst OS (HR, 2.30; 95% CI, 1.76-2.99) (Figure 4).

### Nomogram

**Figure 5.**
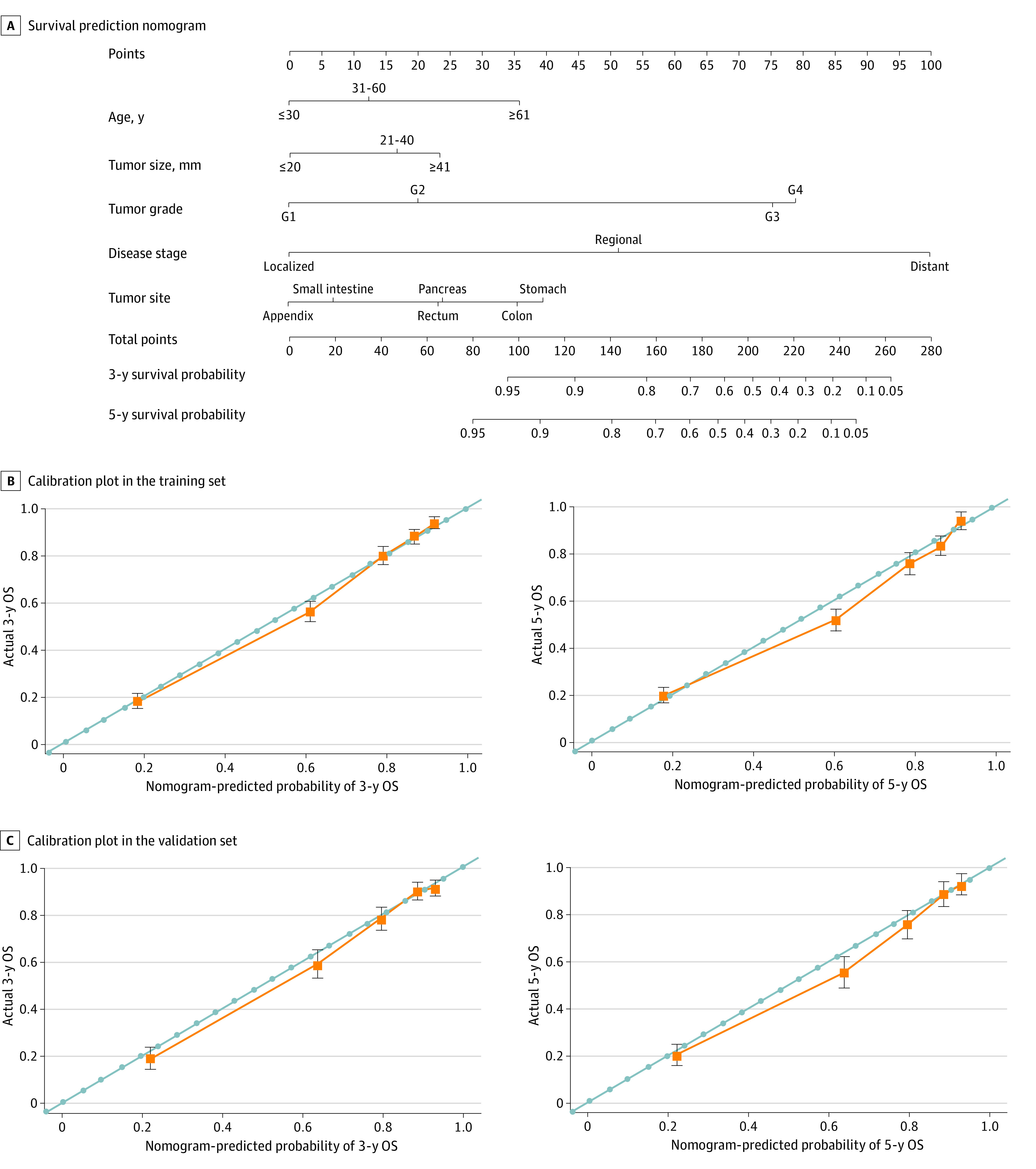
Nomogram to Predict the 3-Year and 5-Year Survival Probabilities of Patients With Gastroenteropancreatic Neuroendocrine Tumors and the Calibration of the Nomogram Using the Training and Validation Sets A, Points for age, tumor size, tumor grade, disease stage, and primary tumor site are obtained by drawing a line upward from the corresponding values to the “Points” line. The sum of the points of these 5 factors is located on the “Total points” line, and a line projected down to the bottom scales determines the probabilities of 3-year and 5-year overall survival (OS). B, Calibration plots of the nomogram for 3-year and 5-year survival probabilities in the training set. C, Calibration plots of the nomogram for 3-year and 5-year survival probabilities in the validation set. The gray line represents the ideal nomogram, and the orange line represents the observed nomogram. The predicted probability of OS by the nomogram is projected onto the x-axis, and the actual OS is projected onto the y-axis. Error bars indicate 95% CIs.

A total of 13 515 patients from all included cohorts were brought into the study according to the model-building requirements (eFigure 1 in the Supplement). Among all eligible patients, 9010 were randomly assigned to the training cohort and 4505 were randomly assigned to the validation cohort. eTable 6 in the Supplement lists the baseline clinicopathologic characteristics; there was no statistical difference between the 2 groups. Based on the training cohort, a nomogram model was constructed by including factors associated with OS, according to the Cox proportional hazards regression model (Figure 5A). Disease stage had the greatest significance, contributing a maximum of 100 points. Tumor grade (79 points), primary tumor site (40 points), age (36 points), and tumor size (24 points) were also individually associated with OS. eTable 7 in the Supplement shows the specific scores for each variable. The nomogram was internally and externally validated. In the training cohort (internal validation) and validation cohort (external validation), the C indexes for OS prediction in the nomogram were 0.893 (95% CI, 0.883-0.903) and 0.880 (95% CI, 0.866-0.894), respectively. Finally, the calibration plots of the nomogram showed consistency between the nomogram-predicted and actual outcomes in the internal (Figure 5B) and external (Figure 5C) validation.

In addition, we further compared the predictive ability of the nomogram and TNM staging through the AUC models of 3-year and 5-year OS rates (eFigure 4 in the Supplement). For the SEER data sets, the AUCs of the nomogram predicting the 3-year and 5-year OS rates were 0.908 and 0.893, respectively, while for the TNM staging, the AUCs were 0.795 and 0.791, respectively. As eFigure 4 in the Supplement shows, the nomogram showed a better predictive ability for survival compared with the TNM staging system.

## Discussion

In this population-based study, we used the large amount of data integrated in the SEER Program to analyze the largest series of GEP-NET cases reported through 2015, with a focus on epidemiologic and prognostic factors. The overall incidence of GEP-NETs showed a persistent increase over 40 years, consistent with the trend noted in early epidemiologic studies.^6,24,25^ Whether the reported increase in the incidence of GEP-NETs is due to underlying biological factors, environmental factors, dietary habits, or the use of certain drugs (such as proton pump inhibitors) remains unknown.^6,26,27^

We analyzed many details about the trends of the incidence of GEP-NETs and found that the trends at each anatomical site have increased, with the largest increases in the stomach and rectum. A steady increase in the incidence of GEP-NETs at these sites may be associated with the increased availability of routine monitoring, cross-sectional imaging, and endoscopy in clinical practice. Although the incidence was increased at all stages in this study, the most significant increase was in the localized stage, which may be due to the increased detection of asymptomatic early-stage disease.^27^ Similarly, the sharp increase in the incidence of G1 GEP-NETs may be associated with the increased awareness and widespread application of the nomenclature and classification of these diseases.^28^ The increasing incidence and prevalence of GEP-NETs underlines the need for more relevant studies to assess the best treatment for these patients.

In this study, we performed a survival analysis using the SEER database and confirmed the significance of age, primary tumor site, tumor grade, and tumor stage at the diagnosis of prior findings in prognoses.^6,7,25^ Our findings are consistent with other studies that patients with colonic or pancreatic NETs appear to have poorer outcomes, whereas patients with rectal or appendiceal NETs have the best prognosis.^7,29^ In our analysis, patients with GEP-NETs with local disease at the time of diagnosis performed better than those with regional and distant disease. This result highlights the importance of early detection and treatment for NETs. For the improvements in the survival of the entire cohort over time, we speculate that nonmetastatic disease–related factors played a major role, and the improvements may also be partly because of the aforementioned changes in incidence, including the increased ratios of relatively more indolent GEP-NETs, such as gastric and rectal NETs. The improvements in survival were also associated with the progress of anticancer therapies, including the emergence and use of targeted therapies.^30,31^

For further exploration of the risk factors for patients with GEP-NETs, we performed multivariable survival analysis using the Cox proportional hazards regression model. We found that age, sex, race and ethnicity, marital status, tumor size, tumor grade, disease stage, and primary tumor site were associated with survival. In addition, the multivariable Cox proportional hazards regression model revealed that disease stage and tumor grade, which showed higher HRs than other included variables, were the most useful indicators of prognosis for patients with GEP-NETs. This finding was consistent with other studies.^32^

Previous studies have demonstrated the predictive ability of nomograms for NETs located in the stomach,^33^ small intestine,^34^ appendix,^35^ and pancreas.^36^ These results indicated that specific and clinically applicable nomograms could accurately estimate the outcome of patients with NETs. In our study, we included GEP-NETs originating from the appendix, colon, rectum, small intestine, stomach, and pancreas as the research object and considered the primary tumor site as an independent prognostic factor. The results showed that the primary site of the GEP-NETs was an important predictor, which was consistent with previous study.^37^ Through multivariable survival analysis, our nomogram included 5 significant prognostic parameters (age, tumor size, tumor grade, tumor stage, and primary tumor site), which could provide patients with GEP-NETs with simple and accurate prognostic predictions. For example, a 75-year-old patient (36 points) with rectal NETs (23 points) and a 3-cm tumor (17 points) with poor differentiation (G3; 75 points) and regional stage (51 points) would have a 3-year survival rate of 50% (202 points) and a 5-year survival rate of 36% (202 points) according to our nomogram. In addition, the predictive ability of the nomogram was also compared with the traditional TNM staging system. Our results demonstrated that the nomogram showed substantially better predictive ability than the TNM staging system. Overall, this simple and effective tool could more accurately evaluate various parameters of GEP-NETs, thereby facilitating clinical decision-making and communication with patients and their families.

### Limitations and Strengths

Our study has several limitations. First, the SEER database might not capture all GEP-NET cases, particularly smaller tumors that may appear benign and may not be registered as malignant neoplasms. For example, data on many small and benign-appearing tumors, such as appendiceal tumors, might not be registered in the SEER database. Therefore, we might actually underestimate the true incidence and prevalence of GEP-NETs. In addition, because the Ki-67 and mitotic index, which are important for the grading of tumors,^38^ are lacking in the SEER database, tumor classification did not take these factors into account. Therefore, it is necessary to carry out large-scale studies to clarify this issue in the future. Finally, in past decades, novel targeted therapies have been used to treat patients with locally advanced or distant metastatic GEP-NETs and have had promising survival benefits in selected cohorts.^39^ The lack of this information might confound the results of survival analysis. However, our study also has several strengths. To our knowledge, this study is one of the largest and most recent explorations of GEP-NETs, and its size and long-term follow-up data have largely made up for the shortcomings and provided comprehensive epidemiologic and survival data on GEP-NETs.

## Conclusions

In this study, the incidence and prevalence of GEP-NETs have continued to increase over 40 years, especially at specific sites (such as the rectum and stomach). Differences were seen in survival rates according to primary tumor site, tumor grade, and tumor stage. However, as diagnosis and treatment progressed, the outcomes generally improved. Furthermore, a novel nomogram, in which tumor stage was the most useful factor, established and validated in the present study, may effectively predict the 3-year and 5-year survival rates of patients with GEP-NETs. It could provide physicians and patients with accurate and useful information and guide the treatment strategy for patients with GEP-NETs.

## Notes

Supplement. **eTable 1.** Histological *ICD* Codes Used to Identify NET Patients From SEER **eTable 2.** Baseline Clinicopathological Characteristics of Gastroenteropancreatic Neuroendocrine Tumors (GEP-NETs) **eTable 3.** Incidence of Gastroenteropancreatic Neuroendocrine Tumors (GEP-NETs) Over Time **eTable 4.** 10-Year and 20-Year Prevalence of Gastroenteropancreatic Neuroendocrine Tumors (GEP-NETs) **eTable 5.** Survival Analysis of Patients With Gastroenteropancreatic Neuroendocrine Tumors (GEP-NETs): Actuarial Survival of GEP-NETs Patients Diagnosed From 1975 to 2015 by Disease Stage and Primary Tumor Site **eTable 6.** Baseline Clinicopathological Characteristics of Patients in the Training and Validation Cohorts **eTable 7.** Detailed Score Assignment for Specific Number/Category of the Parameters Included in the Nomogram **eFigure 1.** A Flowchart of Patient Selection and Study Design **eFigure 2.** Trends in Mean Age at Diagnosis, 3-Year Overall Survival Probabilities, and 5-Year Overall Survival Probabilities According to GEP-NETs Stage (1975-2015) **eFigure 3.** Survival Duration of GEP-NETs by Primary Tumor Site, Age, Stage, and Grade **eFigure 4.** AUC Value of ROC Predicting for the Nomogram and TNM Staging System
