# Supplementary material for: Epidemiologic Trends of and Factors Associated With Overall Survival for Patients With Gastroenteropancreatic Neuroendocrine Tumors in the United States
Source: JAMA Netw Open. 2021 Sep 23;4(9):e2124750. doi: 10.1001/jamanetworkopen.2021.24750 (PMC8461504; doi:10.1001/jamanetworkopen.2021.24750)
Supplement: Supplement. — eTable 1. Histological ICD Codes Used to Identify NET Patients From SEER eTable 2. Baseline Clinicopathological Characteristics of Gastroenteropancreatic Neuroendocrine Tumors (GEP-NETs) eTable 3. Incidence of Gastroenteropancreatic Neuroendocrine Tumors (GEP-NETs) Over Time eTable 4. 10-Year and 20-Year Prevalence of Gastroenteropancreatic Neuroendocrine Tumors (GEP-NETs) eTable 5. Survival Analysis of Patients With Gastroenteropancreatic Neuroendocrine Tumors (GEP-NETs): Actuarial Survival of GEP-NETs Patients Diagnosed From 1975 to 2015 by Disease Stage and Primary Tumor Site eTable 6. Baseline Clinicopathological Characteristics of Patients in the Training and Validation Cohorts eTable 7. Detailed Score Assignment for Specific Number/Category of the Parameters Included in the Nomogram eFigure 1. A Flowchart of Patient Selection and Study Design eFigure 2. Trends in Mean Age at Diagnosis, 3-Year Overall Survival Probabilities, and 5-Year Overall Survival Probabilities According to GEP-NETs Stage (1975-2015) eFigure 3. Survival Duration of GEP-NETs by Primary Tumor Site, Age, Stage, and Grade eFigure 4. AUC Value of ROC Predicting for the Nomogram and TNM Staging System [file jamanetwopen-e2124750-s001.pdf]

Supplementary Online Content

Xu Z, Wang L, Dai S, et al. Epidemiologic trends of and factors associated with overall survival for patients with gastroenteropancreatic neuroendocrine tumors in the United States. *JAMA Netw Open.* 2021;4(9):e2124750. doi:10.1001/jamanetworkopen.2021.24750

eTable 1. Histological *ICD* Codes Used to Identify NET Patients From SEER

eTable 2. Baseline Clinicopathological Characteristics of Gastroenteropancreatic Neuroendocrine Tumors (GEP-NETs)

eTable 3. Incidence of Gastroenteropancreatic Neuroendocrine Tumors (GEP-NETs) Over Time

eTable 4. 10-Year and 20-Year Prevalence of Gastroenteropancreatic Neuroendocrine Tumors (GEP-NETs)

eTable 5. Survival Analysis of Patients With Gastroenteropancreatic Neuroendocrine Tumors (GEP-NETs): Actuarial Survival of GEP-NETs Patients Diagnosed From 1975 to 2015 by Disease Stage and Primary Tumor Site

eTable 6. Baseline Clinicopathological Characteristics of Patients in the Training and Validation Cohorts

eTable 7. Detailed Score Assignment for Specific Number/Category of the Parameters Included in the Nomogram

eFigure 1. A Flowchart of Patient Selection and Study Design

eFigure 2. Trends in Mean Age at Diagnosis, 3-Year Overall Survival Probabilities, and 5-Year Overall Survival Probabilities According to GEP-NETs Stage (1975-2015)

eFigure 3. Survival Duration of GEP-NETs by Primary Tumor Site, Age, Stage, and Grade

eFigure 4. AUC Value of ROC Predicting for the Nomogram and TNM Staging System

This supplementary material has been provided by the authors to give readers additional information about their work.

© 2021 Xu Z et al. *JAMA Network Open.*

eTable 1. Histological *ICD* Codes Used to Identify NET Patients From SEER

**NET Histology**	***ICD* Code**
Islet cell carcinoma	8150/3
Insulinoma	8151/3
Glucagonoma	8152/3
Gastrinoma	8153/3
Mixed islet cell/exocrine adenocarcinoma	8154/3
VIPoma	8155/3
Somatostatinoma	8156/3
Enteroglucagonoma	8157/3
Carcinoid	8240/3
Enterochromaffin cell carcinoid	8241/3
Enterochromaffin-like cell tumors	8242/3
Goblet cell carcinoid	8243/3
Composite carcinoid	8244/3
Adenocarcinoid	8245/3
Neuroendocrine carcinoma	8246/3
Atypical carcinoid tumor	8249/3
Stromal carcinoid	9091/3

© 2021 Xu Z et al. *JAMA Network Open.*

eTable 2. Baseline Clinicopathological Characteristics of Gastroenteropancreatic Neuroendocrine Tumors (GEP-NETs)

Variable	N	%
**Year of diagnosis**		
1975-1991	2741	6.26
1992-1999	3639	8.32
2000-2015	37371	85.42
**Age, y**		
≤ 30	1757	4.02
31-60	23370	53.42
≥ 61	18624	42.57
**Sex**		
Male	21353	48.81
Female	22398	51.19
**Race**		
AI/AN	270	0.62
Asian/P Islander	3207	7.33
Black	7097	16.22
White	31976	73.09
Unknown	1201	2.75
**Marital status**		
Single	7007	16.02
Married	24443	55.87
Sep/Div/Wid	7755	17.73
Unknown	4546	10.39
**Size (mm)**		
≤ 20	7322	16.74
21-40	2969	6.79
≥ 41	2137	4.88
Unknown	31323	71.59
**Grade**		
1	12740	29.12
2	3255	7.44
3	2091	4.78
4	647	1.48
Unknown	25018	57.18
**Disease stage**		
Localized	22502	51.43
Regional	8199	18.74
Distant	9579	21.89
Unstaged	3471	7.93
**Chemotherapy**		
Yes	4442	10.15
No/Unknown	39309	89.85

© 2021 Xu Z et al. *JAMA Network Open.*

Variable	N	%
**Radiation**		
Yes	965	2.21
No/Unknown	42786	97.79
**Surgery**		
Yes	29101	66.52
No/Unknown	14650	33.48
**Primary tumor sites**		
Appendix	3717	8.50
Colon	4010	9.17
Rectum	12532	28.64
Small intestine	12285	28.08
Stomach	4040	9.23
Pancreas	7167	16.38

© 2021 Xu Z et al. *JAMA Network Open.*

eTable 3. Incidence of Gastroenteropancreatic Neuroendocrine Tumors (GEP-NETs) Over Time

Registry	Year	Rate	Lower CI	Upper CI	Number of GEP-NETs cases	Number at risk
SEER 9	1975	1.05	0.9	1.21	117	20,491,638
	1976	0.9	0.76	1.05	114	20,645,967
	1977	1.05	0.9	1.22	131	20,824,399
	1978	0.96	0.82	1.11	137	21,035,770
	1979	1.07	0.92	1.23	144	21,272,552
	1980	1.02	0.87	1.17	121	21,526,796
	1981	0.81	0.68	0.95	107	21,689,241
	1982	0.96	0.82	1.11	123	21,822,466
	1983	0.86	0.73	1	114	21,998,396
	1984	1	0.86	1.15	143	22,197,735
	1985	1.05	0.91	1.2	144	22,423,982
	1986	1.62	1.44	1.81	186	22,644,373
	1987	1.63	1.46	1.82	220	22,872,669
	1988	1.62	1.45	1.8	227	23,111,066
	1989	1.8	1.62	1.99	223	23,349,445
	1990	1.63	1.46	1.81	235	23,657,474
	1991	1.79	1.61	1.98	260	23,998,620
SEER 13	1992	1.81	1.66	1.96	394	35,796,360
	1993	1.88	1.74	2.04	393	36,209,880
	1994	1.79	1.65	1.94	368	36,515,300
	1995	1.95	1.8	2.11	410	36,853,744
	1996	2.03	1.88	2.19	460	37,247,652
	1997	2.21	2.05	2.37	514	37,697,798
	1998	2.36	2.2	2.53	540	38,144,594
	1999	2.49	2.33	2.66	578	38,555,266
SEER 18	2000	2.46	2.34	2.57	1237	78,996,813
	2001	2.59	2.47	2.7	1325	79,867,817
	2002	2.96	2.84	3.08	1586	80,629,975
	2003	3.01	2.89	3.14	1599	81,347,854
	2004	3.11	2.98	3.23	1761	82,055,585
	2005	3.16	3.04	3.29	1723	80,414,394
	2006	3.4	3.27	3.52	1986	83,099,557
	2007	3.55	3.42	3.68	2168	83,810,676
	2008	3.65	3.52	3.78	2233	84,618,783
	2009	3.79	3.66	3.92	2441	85,402,713
	2010	4.03	3.89	4.16	2686	86,151,845
	2011	4.06	3.93	4.2	2801	86,835,341
	2012	4.34	4.21	4.48	2985	87,505,588
	2013	4.65	4.51	4.79	3334	88,122,079
	2014	4.93	4.79	5.07	3558	88,770,355
	2015	5.45	5.31	5.61	4124	89,405,593

© 2021 Xu Z et al. *JAMA Network Open.*

eTable 4. 10-Year and 20-Year Prevalence of Gastroenteropancreatic Neuroendocrine Tumors (GEP-NETs)

Year	20-year duration Prevalence	20-year Count	10-year duration Prevalence	10-year Count
1996	0.00138%	516		
1997	0.00268%	1,016		
1998	0.00401%	1,536		
1999	0.00539%	2,089		
2000	0.00688%	2,689		
2001	0.00834%	3,286		
2002	0.01007%	3,985		
2003	0.01177%	4,680		
2004	0.01359%	5,432		
2005	0.01529%	6,148		
2006	0.01718%	6,952	0.00272%	1,100
2007	0.01926%	7,850	0.00544%	2,216
2008	0.02114%	8,692	0.00801%	3,294
2009	0.02344%	9,716	0.01089%	4,513
2010	0.02587%	10,809	0.01383%	5,781
2011	0.02837%	11,960	0.01685%	7,103
2012	0.03081%	13,101	0.01979%	8,414
2013	0.03354%	14,373	0.02306%	9,881
2014	0.03617%	15,622	0.02617%	11,306
2015	0.03917%	17,047	0.02960%	12,881

© 2021 Xu Z et al. *JAMA Network Open.*

eTable 5. Survival Analysis of Patients With Gastroenteropancreatic Neuroendocrine Tumors (GEP-NETs): Actuarial Survival of GEP-NETs Patients Diagnosed From 1975 to 2015 by Disease Stage and Primary Tumor Site

Tumor Site	Localized				Regional				Distant		
	Median Survival (months)	Survival Rate (%)			Median Survival (months)	Survival Rate (%)			Median Survival (months)	Survival Rate (%)	
		3-Year	5-Year			3-Year	5-Year			3-Year	5-Year
All	NR	98.0	97.2		297	86.1	81.1		34	48.8	38.6
Appendix	NR	98.8	97.4		NR	92.9	88.6		29	40.7	27.4
Colon	402	97.2	96.7		246	75.1	70.3		8	29.8	24.4
Rectum	NR	99.5	99.3		108	67.0	59.7		11	26.5	17.2
Small intestine	NR	96.2	94.8		306	93.3	89.2		96	74.9	63.2
Stomach	NR	96.5	95.5		100	55.6	52.7		9	21.9	17.2
Pancreas	NR	94.2	91.7		130	77.6	68.6		24	40.3	28.6

© 2021 Xu Z et al. *JAMA Network Open.*

eTable 6. Baseline Clinicopathological Characteristics of Patients in the Training and Validation Cohorts

Variables	All patients (n=13515)<br>N (%)		Training set (n=9010)<br>N (%)		Validation set (n=4505)<br>N (%)		*P* value
**Age, y**							< 0.001
≤ 30	732 (5.42)		489 (5.43)		243 (5.39)		
31-60	7144 (52.86)		4755 (52.77)		2389 (53.03)		
≥ 61	5639 (41.72)		3766 (41.80)		1873 (41.58)		
**Sex**							< 0.001
Male	6699 (49.57)		4459 (49.49)		2240 (49.72)		
Female	6816 (50.43)		4551 (50.51)		2265 (50.28)		
**Race**							< 0.001
AI/AN	72 (0.53)		42 (0.47)		30 (0.67)		
Asian/P Islander	994 (7.35)		641 (7.11)		353 (7.84)		
Black	1984 (14.68)		1325 (14.71)		659 (14.63)		
White	10220 (75.62)		6830 (75.80)		3390 (75.25)		
Unknown	245 (1.81)		172 (1.91)		73 (1.62)		
**Marital status**							< 0.001
Single	2518 (18.63)		1655 (18.37)		863 (19.16)		
Married	7780 (57.57)		5204 (57.76)		2576 (57.18)		
Sep/Div/Wid	2264 (16.75)		1486 (16.49)		778 (17.27)		
Unknown	953 (7.05)		665 (7.38)		288 (6.39)		
**Grade**							< 0.001
1	9690 (71.70)		6470 (71.81)		3220 (71.48)		
2	2341 (17.32)		1555 (17.26)		786 (17.45)		
3	1139 (8.43)		748 (8.30)		391 (8.68)		
4	345 (2.55)		237 (2.63)		108 (2.40)		
**Disease stage**							< 0.001
Localized	6986 (51.69)		4658 (51.70)		2328 (51.68)		
Regional	3675 (27.19)		2423(26.89)		1252 (27.79)		
Distant	2854 (21.12)		1929 (21.41)		925 (20.53)		
**Chemotherapy**							< 0.001
Yes	1453 (10.75)		995 (11.04)		458 (10.17)		
No/Unknown	12062 (89.25)		8015 (88.96)		4047 (89.83)		
**Radiation**							< 0.001
Yes	322 (2.38)		221 (2.45)		101 (2.24)		
No/Unknown	13193 (97.62)		8789 (97.55)		4404 (97.76)		
**Surgery**							< 0.001
Yes	12049 (89.15)		8021 (89.02)		4028 (89.41)		
No/Unknown	1466 (10.85)		989 (10.98)		477 (10.59)		
**Primary tumor site**							< 0.001
Appendix	1584 (11.72)		1051(11.66)		533 (11.83)		
Colon	1260 (9.32)		841 (9.33)		419 (9.30)		
Rectum	2370 (17.54)		1605 (17.81)		765 (16.98)		
Small intestine	4341 (32.12)		2861 (31.75)		1480 (32.85)		
Stomach	1020 (7.55)		682 (7.57)		338 (7.50)		

© 2021 Xu Z et al. *JAMA Network Open.*

Pancreas	2940 (21.75)		1970 (21.86)		970 (21.53)		

© 2021 Xu Z et al. *JAMA Network Open.*

eTable 7. Detailed Score Assignment for Specific Number/Category of the Parameters Included in the Nomogram

Prognostic factors		
Variable	Category	Score
Age (year)	≤ 30	0
	31-60	12
	≥ 61	36
Size (mm)	≤ 20	0
	21-40	17
	≥ 41	24
Tumor grade	G1	0
	G2	20
	G3	75
	G4	79
Disease stage	Localized	0
	Regional	51
	Distant	100
Tumor site	Small intestine	0
	Appendix	7
	Rectum	23
	Pancreas	24
	Colon	36
	Stomach	40
3-year survival		
Total score	3-year survival probability	
	0.05	263
	0.1	252
	0.2	237
	0.3	225
	0.4	214
	0.5	202
	0.6	190
	0.7	175
	0.8	156
	0.9	125
	0.95	95
5-year survival		
Total score	5-year survival probability	
	0.05	248
	0.1	237
	0.2	222
	0.3	210
	0.4	199
	0.5	187
	0.6	175
	0.7	160

© 2021 Xu Z et al. *JAMA Network Open.*

Prognostic factors		
Variable	Category	Score
	0.8	141
	0.9	110
	0.95	80

© 2021 Xu Z et al. *JAMA Network Open.*

eFigure 1. A Flowchart of Patient Selection and Study Design. GEP-NETs, gastroenteropancreatic neuroendocrine tumors; ICD-O-3, International Classification of Diseases for Oncology, 3rd Edition; SEER, the Surveillance, Epidemiology, and End Results dataset.

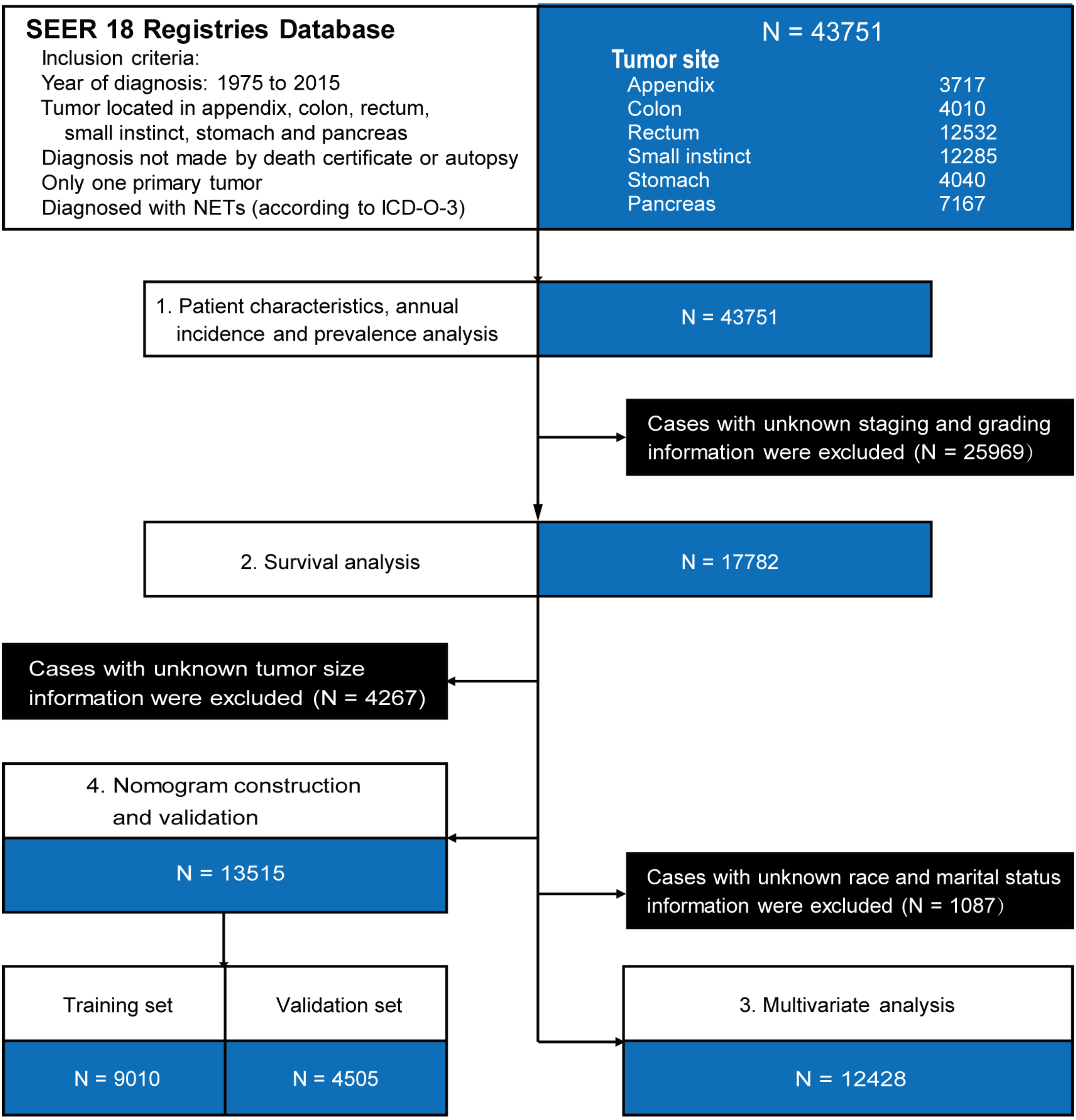

© 2021 Xu Z et al. *JAMA Network Open.*

eFigure 2. Trends in (A) Mean Age at Diagnosis, (B) 3-Year Overall Survival Probabilities, and (C) 5-Year Overall Survival Probabilities According to GEP-NETs Stage (1975-2015). GEP-NETs, gastroenteropancreatic neuroendocrine tumors.

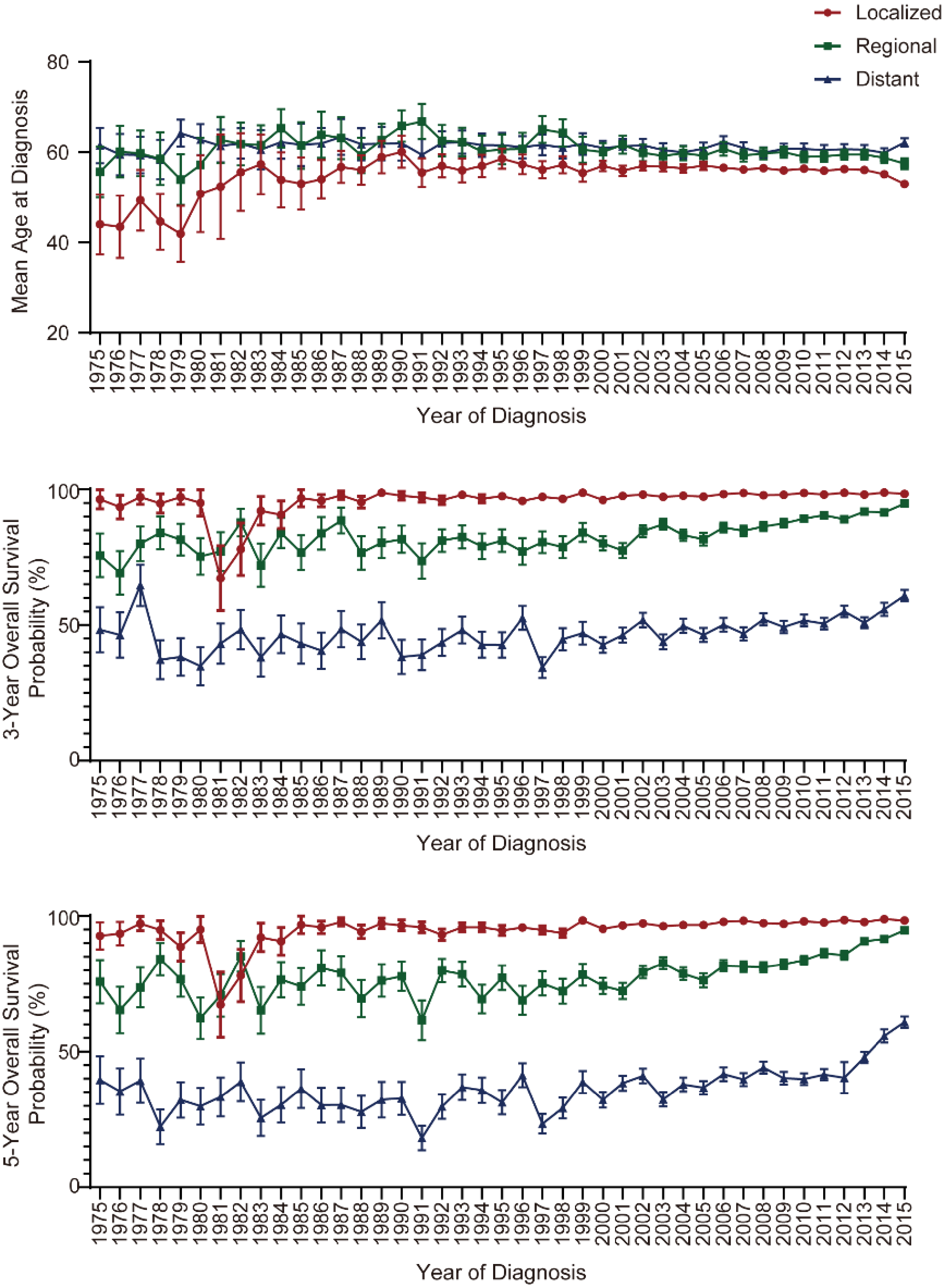

© 2021 Xu Z et al. *JAMA Network Open.*

eFigure 3. Survival Duration of GEP-NETs by (A) Primary Tumor Site, (B) Age, (C) Stage, and (D) Grade. Median survival durations are presented in months (with 95% CIs). GEP-NETs, gastroenteropancreatic neuroendocrine tumors; CI, confidence interval.

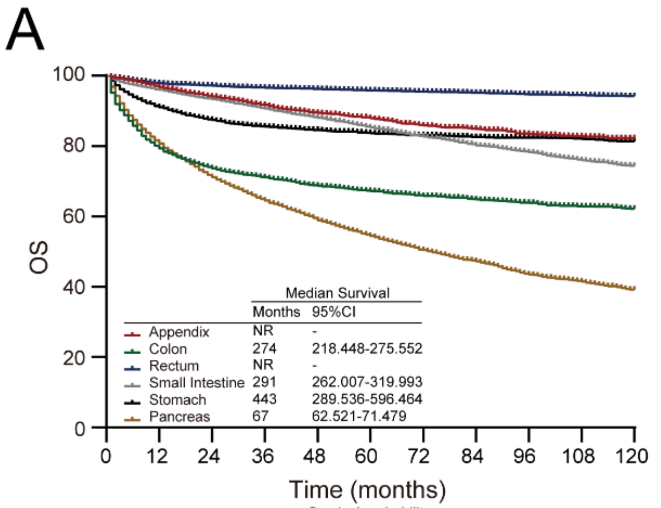

Survival probability

Appendix	1	0.96	0.94	0.91	0.89	0.88	0.85	0.84	0.83	0.82	0.82
Colon	1	0.75	0.70	0.67	0.65	0.64	0.62	0.61	0.60	0.59	0.59
Rectum	1	0.98	0.97	0.96	0.96	0.96	0.95	0.95	0.94	0.94	0.94
Small Intestine	1	0.95	0.92	0.89	0.87	0.84	0.81	0.79	0.77	0.75	0.73
Stomach	1	0.89	0.85	0.84	0.83	0.82	0.81	0.81	0.81	0.80	0.80
Pancreas	1	0.78	0.68	0.62	0.57	0.52	0.48	0.45	0.42	0.40	0.38

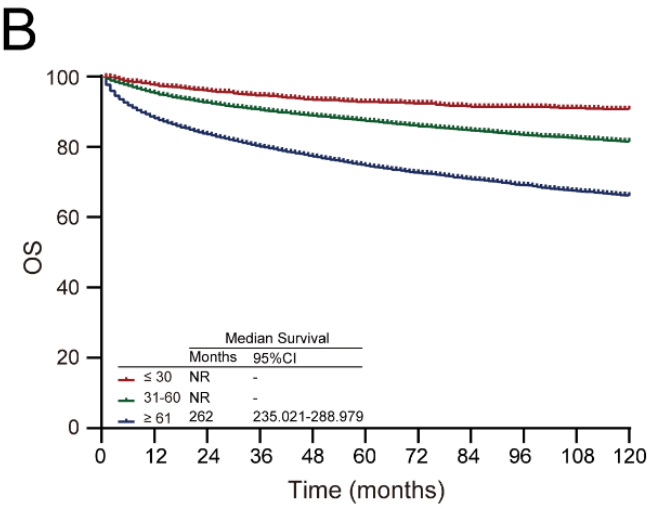

Survival probability

≤ 30	1	0.97	0.96	0.94	0.93	0.93	0.92	0.91	0.91	0.91	0.90
31-60	1	0.95	0.92	0.90	0.88	0.87	0.85	0.84	0.83	0.82	0.81
≥ 61	1	0.85	0.81	0.77	0.75	0.72	0.70	0.68	0.67	0.65	0.64

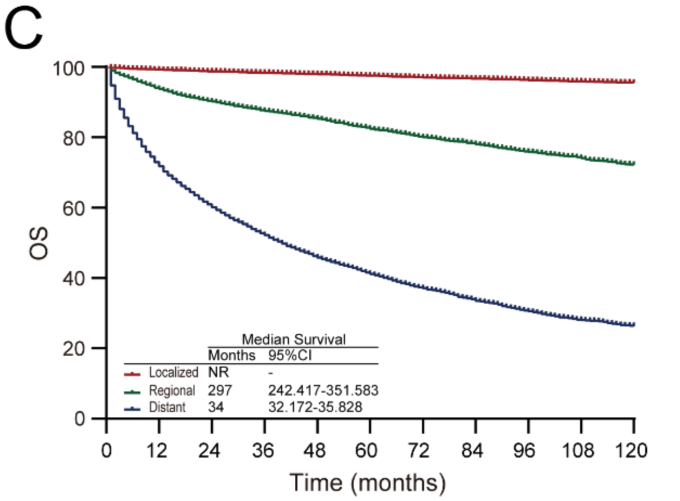

Survival probability

Localized	1	0.99	0.98	0.98	0.98	0.97	0.97	0.96	0.96	0.96	0.95
Regional	1	0.92	0.89	0.86	0.84	0.81	0.79	0.77	0.75	0.73	0.71
Distant	1	0.67	0.56	0.49	0.43	0.39	0.35	0.31	0.29	0.26	0.25

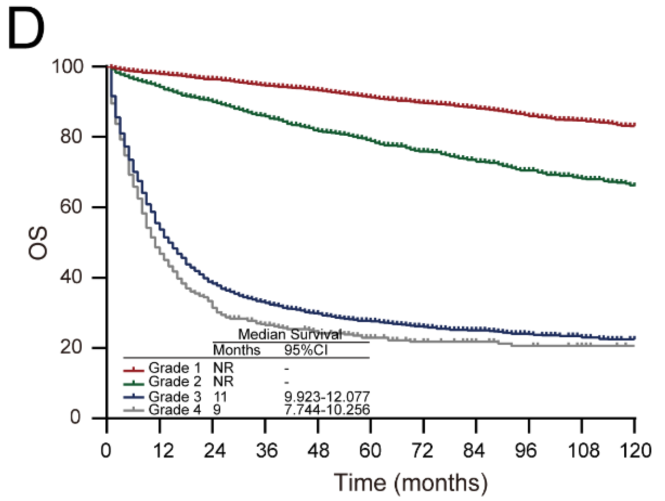

Survival probability

Grade Ⅰ	1	0.98	0.96	0.94	0.93	0.91	0.89	0.88	0.86	0.84	0.83
Grade Ⅱ	1	0.93	0.89	0.85	0.81	0.78	0.75	0.72	0.70	0.67	0.65
Grade Ⅲ	1	0.48	0.35	0.30	0.27	0.25	0.23	0.23	0.22	0.21	0.20
Grade Ⅳ	1	0.42	0.28	0.24	0.22	0.21	0.20	0.20	0.19	0.19	0.19

© 2021 Xu Z et al. *JAMA Network Open.*

eFigure 4. AUC Value of ROC Predicting for the Nomogram and TNM Staging System. Area under the curves of the two models to predict 3-year survival rates (A) and 5-years survival rates (B) using the SEER datasets. AUC, area under the curve; ROC, receiver operating characteristic; SEER, the Surveillance, Epidemiology, and End Results dataset.

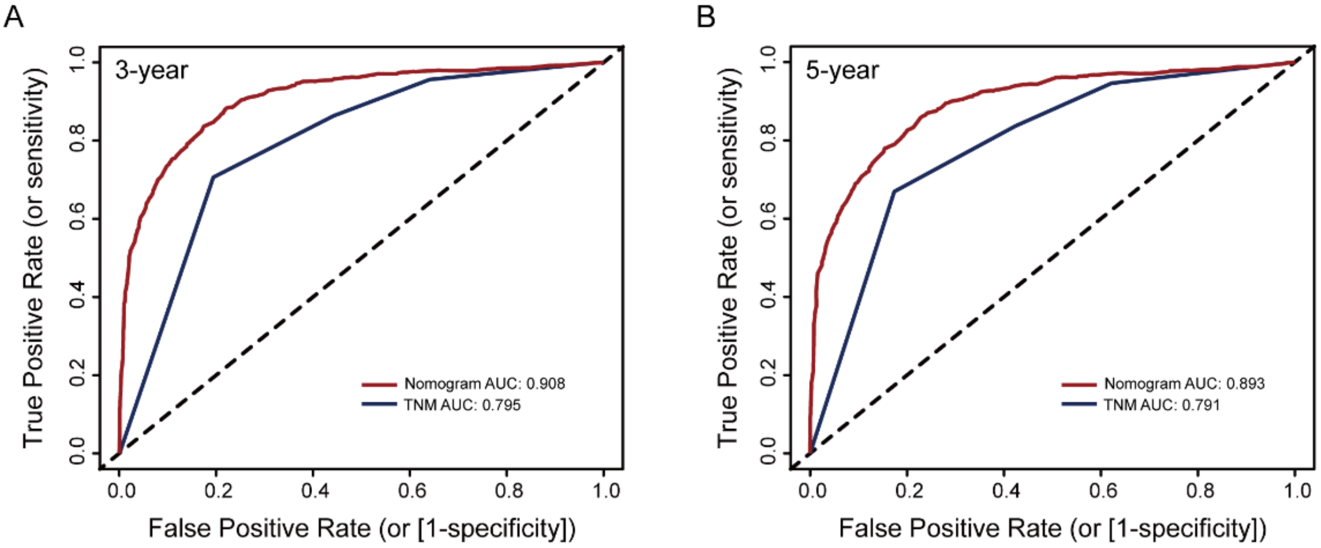

© 2021 Xu Z et al. *JAMA Network Open.*
